# Commentary: Pupil old/new effects reflect stimulus encoding and decoding in short-term memory

**DOI:** 10.3389/fpsyg.2017.00277

**Published:** 2017-02-28

**Authors:** Alex Kafkas, Daniela Montaldi

**Affiliations:** Memory Research Unit, Division of Neuroscience and Experimental Psychology, School of Biological Sciences, University of ManchesterManchester, UK

**Keywords:** pupil response, recognition memory, familiarity, recollection, old/new effect

The pupil response has emerged as a measure of long-term memory encoding (Kafkas and Montaldi, 2011, 2015a; Papesh et al., 2012) and retrieval (Võ et al., 2008; Kafkas and Montaldi, 2012, 2015b) in recognition memory tasks. At retrieval, the pupil dilates more for old than new stimuli; the pupil old/new effect (Võ et al., 2008). Moreover, pupil response patterns have been found to discriminate between familiar and recollected stimuli (Otero et al., 2011; Kafkas and Montaldi, 2012). Familiarity and recollection are kinds of memory that support an “old” response in a recognition memory task; a recollected stimulus brings to mind associative information from the encoding event, while a familiar stimulus does not. The increased pupil dilation characterizing old stimuli does not just reflect the recovery of associative information (i.e., recollection) as familiar stimuli also produce larger dilation patterns relative to new (Kafkas and Montaldi, 2015b).

Recently, Brocher and Graf (2016) replicated the pupil old/new effect in a series of experiments manipulating variables that have previously been shown to differentially modulate ERP components assumed to be the signatures of familiarity and recollection. Unlike previous research, however, they did not measure familiarity and recollection performance, while still concluding that pupil responses do not distinguish between these forms of memory. Here we discuss several issues of concern regarding terminology, methodology and logic of data interpretation. We note two critical errors in terminology and one critical error of reporting on a published paper. First, Borcher and Graf refer to their retrieval phase, which is in fact a recognition task, as the “recall phase.” This distinction is absolutely critical to memory theory; “recall” involves bringing to mind information, and does not describe the old/new recognition paradigm where such retrieval may occur but is certainly not necessary. Second, as the title suggests, the authors argue that their findings “are compatible with the view that pupil old/new effects reflect strength of memory traces in short-term memory” (Brocher and Graf, 2016, p. 1832), however, their reported experiments only made use of long-term memory paradigms. Short-term memory, characterized by limited capacity and short-term retention was not investigated in their study so the use of the term is inaccurate and misleading. More specifically, although their experiments are characterized by relatively short periods of sustained encoding of a series of items followed immediately by testing, the timing is not consistent with a short-term memory explanation. A short-term memory task, requires that items are tested immediately after one, or at most, a very few presentations, but in the Brocher and Graf tasks, a block of 40 stimuli, which far exceeds the capacity of short-term memory, was encoded before test. This is not to say that short-term memory manipulations can have no potential effect on the pupil response, they may, but Brocher and Graf have not demonstrated this, and their effects can only be attributed to long-term memory. Moreover, their interpretation fails to acknowledge the rapidly growing literature replicating findings showing the sensitivity of the pupil response to *long-term memory* (Võ et al., 2008; Kafkas and Montaldi, 2011, 2012, 2015a,b; Otero et al., 2011; Papesh et al., 2012). Thirdly, we would like to clarify a point made in relation to our publication (Kafkas and Montaldi, 2015b) which Brocher and Graf discuss. They state that when a simpler decision was made by participants, in Kafkas and Montaldi (2015b), the pupil old/new effect was not replicated. To the contrary, our finding was quite the opposite; we clearly reported that the sensitivity of the pupil to old/new responses was maintained with simple decisions, and was therefore independent of task complexity.

Our final concern relates to a set of assumptions, or inferences, made by Brocher and Graf, which although unjustified, are used to support much of what they conclude regarding recollection and familiarity. The authors overarching assumption is that if a cognitive variable differentially affects the ERP components of familiarity and recollection, then it should also differentially affect the pupil responses. This is a very flawed misconception based on a set of three unsupported assumptions: (a) equivalence between the sources of the ERP signals and pupil response signals, (b) that two distinct ERP components (frontal and parietal) incontrovertibly reflect familiarity and recollection memory, and (c) that the manipulated cognitive variables modulate familiarity and/or recollection in a reliable way across all tasks. In relation to the first assumption, there is no reason to assume equivalence between the ERP and pupil response signals, which are controlled by different neurophysiological systems (e.g., Beatty and Lucero-Wagoner, 2000 for pupil response; Buzsáki et al., 2012 for ERP). EEG measures cortical activity while the pupil response measures autonomic (not central) nervous system activity and provides a summative outcome indicator of underlying cognitive processing whose source cannot be spatially localized. Regarding the second assumption, the specificity of the presumed familiarity and recollection ERP components remains under investigation (as Brocher and Graf themselves imply) and therefore to use them as strong proxies for different forms of memory is unjustified. Indeed this type of reverse inference has been heavily criticized, even with fMRI, where spatial resolution is orders of magnitude better (e.g., Poldrack, 2006). The third assumption involves a similar inference, and is that manipulations of lexicality, valence and word frequency elicit differential effects on familiarity and recollection that are robust, reliable and predictable across all tasks and, when manipulated, can be used to interpret pupil effects as reflecting either recollection, familiarity or both. The logic is clear, but unfortunately, the evidence on which this assumption is based is not strong; first because the relationship between such factors and memory performance or ERPs varies across studies and task demands (see Gardiner and Java, 1990; but see Perfect and Dasgupta, 1997) and second because some of the evidence the authors draw on is very tenuous. For example, their hypothesis in the valance experiment (Experiment 2) is based on the flawed argument that because two studies (Van Strien et al., 2009; Xu et al., 2015) have found stronger old/new familiarity ERP signals (FN400) for either positive or negative pictures, but equally sensitive old/new recollection ERP signals (LPC) for these stimuli, the lack of difference in the pupil old/new effect for positive and negative pictures in their Experiment 2 would imply that the pupil signal reflects recollection. There is no justification for this reverse inference, which is highly misguided.

In conclusion, while Brocher and Graf “challenge the view that pupil old/new effects can be directly mapped onto familiarity and/or recollection” (Brocher and Graf, 2016, p. 1829), we argue that using ERP signals as proxies for behavioral responses, is misleading and falls far short of what is needed to establish the conclusions the authors wish to draw, as does the dependence on predictions derived from ERP old/new effects that have no bearing on pupil activity. We suggest that the minimum requirement to establish this challenge, especially considering the substantial evidence to the contrary, involves directly testing recollection and familiarity while collecting pupil data, as done in other studies. If this were carried out, the authors' conclusions would not be dependent on any of the unsubstantiated assumptions and inferences we highlight.

## Author contributions

AK and DM wrote the paper. Both authors approved the manuscript for publication.

## Funding

AK is supported by the Wellcome Trust (Grant number 094597).

### Conflict of interest statement

The authors declare that the research was conducted in the absence of any commercial or financial relationships that could be construed as a potential conflict of interest.
